# Development of a Virus-Based Reporter System for Functional Analysis of Plant rRNA Gene Promoter

**DOI:** 10.3389/fmicb.2021.637347

**Published:** 2021-02-12

**Authors:** Li Xu, Zhiying Li, Sheng Wang

**Affiliations:** ^1^Key Laboratory of Ministry of Education for Protection and Utilization of Special Biological Resources in Western China, School of Life Science, Ningxia University, Yinchuan, China; ^2^Key Laboratory of Modern Molecular Breeding for Dominant and Special Crops in Ningxia, School of Life Science, Ningxia University, Yinchuan, China

**Keywords:** rRNA gene promoter, *Nicotiana benthamiana*, viral vector, transient expression, tomato bushy stunt virus, species-specificity

## Abstract

Reporter gene-based expression systems have been intensively used in plants for monitoring the activity of gene promoters. However, rRNA transcripts are unable to efficiently express a reporter gene due to a lack of a 5' cap. Because of this obstacle, plant rRNA gene promoters are less well characterized to this day. We developed a virus-based reporter system to characterize the *Nicotiana benthamiana* rRNA (*Nb*rRNA) gene promoter. The system utilizes the cap-independent translation strategy of viral genomic mRNA and uses the virus-expressed green fluorescent protein (GFP) as an indicator of the rRNA gene promoter activity in virus-infected plants. Based on the reporter system, some characteristics of the *N. benthamiana* rRNA gene promoter were revealed. The results showed that the strength of the *Nb*rRNA gene promoter was lower than that of the cauliflower mosaic virus (CaMV) 35S promoter, a well-characterized polymerase II promoter. The sequences between −77 and +42 are sufficient for the *Nb*rRNA gene promoter-mediated transcription and the *Nb*rRNA gene promoter may lack the functional upstream control element (UCE). Interestingly, *Nb*rRNA gene promoter activity was increased when the 35S enhancer was introduced. An intron-excision mediated assay revealed that the *Nb*rRNA gene promoter can be inefficiently used by RNA polymerase II in *N. benthamiana* cells. This virus-based reporter system is easier to operate and more convenient when compared with the previously Pol I promoter assays. And it offers a promising solution to analyzing the functional architecture of plant rRNA gene promoter.

## Introduction

Transcription of nuclear DNA to RNA in eukaryotic cells is carried out by at least three distinct RNA polymerases (Roeder and Rutter, 1969). Each of the RNA polymerases is responsible for the transcription of certain genes (Cramer et al., 2008). RNA polymerase I (Pol I) transcribes the genes encoding three (18S, 5.8S, and 28S) ribosomal RNAs (rRNAs), which together with the 5S rRNA synthesized by RNA polymerase III (Pol III), constitute the primary component of the ribosome, thereby controlling the overall level of biological protein synthesis in living cells (Russell and Zomerdijk, 2006). Thus, rRNAs are regarded as one of the major regulators of the destiny of a cell (Sharifi and Bierhoff, 2018). The control of rRNA transcription is one of the most enduring issues in molecular biology (Kuhn et al., 2007) and rRNA gene promoters are one of the subjects needing to be intensively investigated.

In eukaryotes, rRNA gene promoters are located in the intergenic spacers (IGS) that separate the numerous rRNA gene transcription units (Grummt, 2003). In animals, rRNA gene promoters generally consist of a core promoter sequence, which directs basal transcription and contains sequences from about −40 to +10 relative to the transcription initiation site (TIS), and an upstream control element (UCE), which strengthens rRNA transcription and typically extends to approximately −150 (Russell and Zomerdijk, 2005). Animal rRNA gene promoters are usually not functional in heterologous species and share little sequence similarity between species (Mishima et al., 1982). Unlike animals, the consensus sequence motif TATATA(A/G)GGG around TIS is highly conserved in plants (Doelling and Pikaard, 1996). The sequences between −55/−33 and +6 suffice to mediate transcription initiation of rRNA genes in *Arabidopsis thaliana* (Doelling and Pikaard, 1995). In common with animals, plant Pol I-dependent rRNA transcription was proved to be species-specific in one case (Fan et al., 1995). However, the data obtained from another study showed that the foreign rRNA gene promoter can be used by the host Pol II in an inefficient way and with an altered initiation site (Doelling and Pikaard, 1996).

Although the IGS of rDNAs has been identified in a variety of plant species (Poczai and Hyvönen, 2010), the roles of rRNA gene promoters are not well-characterized. Unlike mRNAs, rRNAs lack a 5' cap. Therefore, they cannot be efficiently translated, whether *in vivo* or *in vitro* (Doelling et al., 1993). Consequently, the traditionally used reporter gene-based assay, which is widely used for the analysis of the differential activity of RNA polymerase II promoter, cannot be used to measure the rRNA gene promoter activity in plants. Instead, the rRNA transcription levels must be measured directly by other more complex assays. Otherwise, a large number of endogenous rRNA genes might compete with cloned rRNA genes after they were transfected into cells, resulting in low template utilization (Doelling and Pikaard, 1995). Doelling and Pikaard (1995) proposed that those technical obstacles are one of the biggest problems for studying cloned rRNA gene promoters.

*Tomato bushy stunt virus* (TBSV) belongs to the genus *Tombusvirus* within the family *Tombusviridae* (Yamamura and Scholthof, 2005). The genome of TBSV is composed of a positive-sense single-stranded RNA (+ssRNA), which encodes five open reading frames (ORFs). Translation of those ORFs adopts a cap-independent strategy, which involves an RNA-RNA interaction between untranslated regions (UTRs) of the TBSV RNAs (Fabian and White, 2004). We previously developed a novel non-fusion TBSV-CP replacement vector under the control of the Pol II promoter (35S), which efficiently expresses green fluorescent protein (GFP) *via* viral subgenomic mRNA1 (sg mRNA1) in tobacco plants (Zhang et al., 2020). Theoretically, the translation strategy adopted by TBSV RNAs could be applied to a reporter system for circumventing the need of Pol I transcripts for a cap and conveniently measuring the activity of plant rRNA gene promoter. Moreover, the replication of viral genomic mRNA might conquer the problem of low template utilization.

To test the potential of the virus-based reporter system for functional analysis of plant rRNA gene promoters, a GFP-expressing TBSV-based viral vector was fused downstream of the rRNA gene promoter of the model plant *Nicotiana benthamiana*. Agroinfiltration (*Agrobacterium tumefaciens*-mediated approach) was employed to deliver the viral vectors into *N. benthamiana* leaves. The promoter activity was estimated by measuring the accumulation of recombinant GFP in the agroinfiltrated leaves. Through this virus-mediated reporter system, the characteristic of *N. benthamiana* rRNA gene promoter was analyzed. The results suggest that the established system is suitable for a detailed exploration of the structural architecture of plant rRNA gene promoters.

## Materials and Methods

### Plant Materials and Growth Conditions

The seeds of *N. benthamiana* were planted in 12.5 cm (in diameter) plastic pots filled with the sterilized soils. After germination, the young seedlings were maintained under conditions at 22–25°C with 65% relative humidity and 16 h of light per day. All plants were well-watered when required and fed with standard Hoagland’s culture solution once a week.

### Construction of *Nb*rRNA Gene Promoter-Based TBSV Vectors

All TBSV viral cDNA constructs were created by *in vitro* DNA synthesis and conventional restriction enzyme-mediated cloning method.

For the construction of the 5' deletion constructs of the *Nb*rRNA gene promoter (GenBank accession no. KC352713.1), the various 5' deleted promoter fragments were synthesized *in vitro* with *Apa*I at 5' end and *Pvu*I at 3'end (GenScript, China). The fragments were then subcloned into the corresponding restriction enzyme sites of the Pol II-G vector, which is a non-fusion GFP-expressing TBSV construct under the control of the 35S promoter (Zhang et al., 2020). For clarity, these constructs were named according to their deletion endpoints, −498 (containing sequences −498 to the transcription start site, +1), −251 (−251 to +1), −125 (−125 to +1) and −77 (−77 to +1).

To obtain −77+42 construct, the 407 bp long fragment, which successively contained *Apa*I restriction enzymes site, *Nb*rRNA gene promoter (−77 to +42), hammerhead ribozyme, the 230 nts of 5'-UTR of TBSV genomic RNA (having a *Pvu*I restriction enzymes site at its 3' end), were synthesized and substituted for the *Apa*I-*Pvu*I digested fragment of Pol II-G as described above.

To create two constructs with enhancer sequences, the sequence of Pol I enhancer from yeast (the *Eco*RI-*Hpa*I fragment) and an enhancer sequence of 35S promoter (−343 to −90) were placed at 5' end of 407 bp long fragment of −77+42, respectively. The designed sequences were then synthesized and were separately cloned into the *Apa*I and *Pvu*I sites of the Pol II-G vector to generate E1−77+42 and E2−77+42.

Two derivative constructs of E2−77+42 were achieved by site-directed mutagenesis. Changing A to T and G to C in the promoter region of E2−77+42 at position +15 to +42 generated the E2−77+42 mD construct. Substituting T for C at −3 in the promoter region of E2−77+42 generated the E2−77+42mTA construct.

For the generation of constructs with introns, introns ranging in size from 109 to 159 nt were selected from the *A. thaliana* genome. The intron insertion sites in the viral RNA-dependent RNA polymerase (RdRp) were selected that met the canonical consensus sequences of AG/GT. Nine positions were chosen (position corresponding to TBSV cherry sequence, GenBank accession no. NC_001554) for intron insertion: 1, nt 581; 2, nt 784; 3, nt 939; 4, nt 1,135; 5, nt 1,372; 6, nt 1,581; 7, nt 1,880; 8, nt 2,137; and 9, nt 2,370. The designed sequences were then synthesized and were separately cloned into the *Pvu*I and *Nhe*I sites of the previously constructed vectors to generate corresponding intron-containing viral vectors.

### Agroinfiltration of *N. benthamiana* Leaves

The recombinant plasmids were transformed into competent *A. tumefaciens* cells of strain GV3101 according to procedures of the freeze-thaw method and then transformed cells were plated on LB agar plate with appropriate antibiotics (kanamycin, rifampicin, and gentamycin, 50 μg/ml for each) for plasmid selection. Colony PCR was used for identifying the positive recombinant colony. Selected *A. tumefaciens* colony was transferred into 4 ml of LB liquid medium containing appropriate antibiotics (kanamycin, rifampicin, and gentamycin, 50 μg/ml for each) and then incubated (28°C and 250 rpm shaking) for 24 h. After adding another 100 ml of LB medium (containing 200 μM acetosyringone), the cultured cells were keep growing overnight under the same conditions. Cells harvested by centrifugation at 4,000 *g* were suspended and adjusted to an OD_600_ (Optical Density at 600 nm) of 1.0 by using MES buffer (pH 5.6, 10 mM MgCl_2_, and 200 μM acetosyringone). The cell suspensions were put into the dark chamber and allowed to stand at room temperature for 2 h just before infiltration. After treatment for 2-h standing, the suspensions were infiltrated into *N. benthamiana* leaves *via* a needleless syringe.

In one experiment, 15–20 seedlings and three leaves of each seedling were infiltrated. Three independent experiments were performed. The infiltrated leaves were then sampled at 4, 6, and 8 days post-inoculation (dpi). Every sample consisted of about 15 leaf disks that were cut out from inoculated leaves. The harvested leaves were stored in a −80°C refrigerator for later analysis. All samples were analyzed in triplicate.

### GFP Imaging

Green fluorescent protein signals were observed in infiltrated leaves under irradiation by a hand-held UV-lamp. And all of the pictures of images were taken in the darkened room by the use of a digital camera.

### Protein Extraction

Leaf tissue samples were frozen in liquid nitrogen and ground to a fine powder. A total of 0.1 g of leaf powder was then mixed with 400 μl of extraction buffer (50 mM Tris-HCl, 150 mM NaCl, 10 mM EDTA, pH 8.0). The tissue homogenates were centrifuged at 12,000 *g* for a total of 15 min at 4°C. The supernatant containing total soluble proteins was transferred to a new tube and then was used in Western Blot or ELISA analysis immediately.

### SDS-Page and Western Blot Analysis

Protein samples were heated at 95°C for about 5 min and then loaded on a 12% polyacrylamide gel [6.8 × 8.6 cm (length × width), 0.75 mm spacer set] for protein separation. After electrophoresis (1 h at 180 V), the gel was stained overnight in the solution of Coomassie Brilliant Blue G-250 with gently shaking. For Western blot assays, a semi-dry electrophoresis transfer unit (Bio-Rad) was used to transfer the proteins from SDS-gel to a 0.45 μm NC membrane (Sigma-Aldrich). Following an overnight-block with phosphate-buffered saline (PBS) made 5% non-fat dry milk, strips from the blot were developed by rabbit polyclonal anti-GFP antibody (Abcam) with 1:1,000 dilutions, next by 1:5,000 goat anti-rabbit HRP conjugated secondary antibody (Sigma-Aldrich). Specific protein bands were visualized after the treatment of the strips with ECL-solution (GE Healthcare).

### GFP Quantification Assay

ELISA assay was applied to measure the accumulation of recombinant GFP in the inoculated leaves. ELISA plate filled with 100 μl of protein samples per well was incubated overnight at 4°C for antigen coating. Following washing procedures (three times and 5 min for each with PBS), 100 μl of 1:10,000 rabbit polyclonal anti-GFP antibody (in PBS) was added to each well of the plate, and then the plate was put into an incubator at 37°C for 2 h. Next, 100 μl of 1: 5,000 secondary antibody (HRP conjugated goat anti-rabbit antibody, in PBS) was added to the carefully washed plate. After a 2-h incubation and a washing step, each plate-well was re-filled with 100 μl of tetramethylbenzidine (TMB) substrate solution (Solarbio) and incubated for 15–30 min. After that, 100 μl of phosphoric acid (1.0 M) was pipetted into wells to stop the reaction. A microplate reader (Bio-Rad) was employed to read optical density (OD) values at a wavelength of 450 nm. Meanwhile, *Escherichia coli*-produced GFP (BioVision) was used to generate an ELISA standard curve, and then the standard curve was utilized for calculating the GFP accumulation in the collected leaf samples. The protein samples from the empty vector infiltrated leaves were used as a negative control. The observed values from three independent experiments were performed with Student’s *t*-test and a value of *p* < 0.05 was considered significant.

### RNA Extraction and Reverse Transcription PCR

Total RNAs of *N. benthamiana* were extracted from 0.1 g of fresh tissue using the RNAprep Pure Plant Kit (TIANGEN, China) according to the manufacturer’s instructions. Prepared total RNA (10 ng) was used to synthesize cDNAs by using a first-strand cDNA synthesis kit (TransGen Biotech, China). The primer pair was 46intr-F (5'-GTAACACAGATCAATCGAG-3') and 46intr-R (5'-TGGATTCCATATGCCGTAG-3'). The predicted size of the PCR product with or without introns was 919 and 541 bp, respectively.

## Results

### Effect of the Sequences Located Upstream of TIS on *Nb*rRNA Gene Promoter Activity

**Figure 1:**
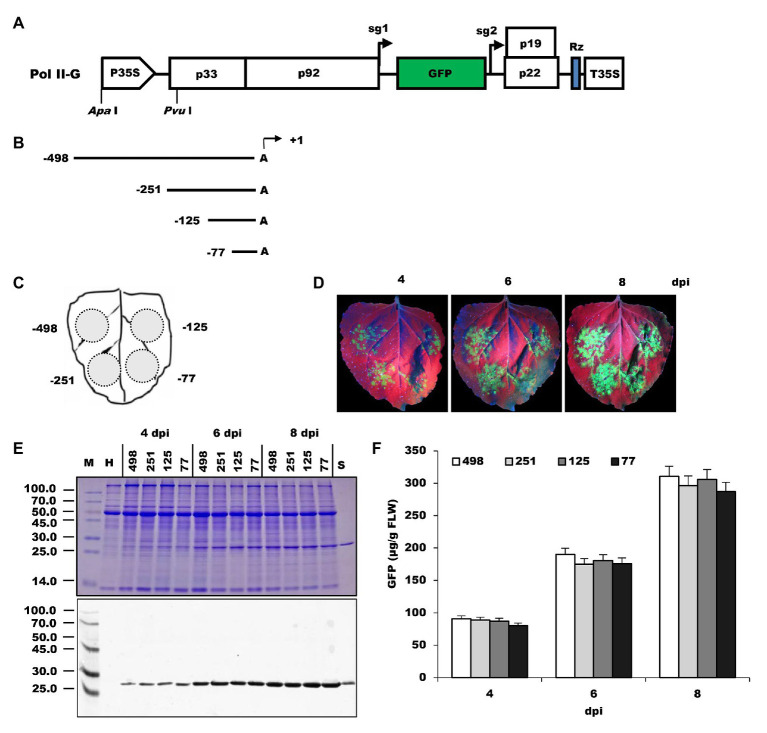
Comparison of *Nb*rRNA gene promoter deletion constructs for their ability to express GFP in *N. benthamiana* leaves. **(A)** Pol II-G, a GFP-expressing *tomato bushy stunt virus* (TBSV)-based viral vector under control of 35S promoter. It was used as a backbone to develop the deletion constructs of the *N. benthamiana* polymerase I promoter. P35S, 35S promoter; p33 and p92, the gene coding replication proteins; sg1 and sg2, the transcription initiation sites for subgenomic mRNAs 1 and 2 indicated by right-angled arrows; GFP, enhanced green fluorescent protein gene; p19, silencing suppressor gene; p22, cell-to-cell movement protein (MP) gene; Rz, ribozyme; T35S, CaMV polyA signal sequence/terminator; *Apa*I and *Pvu*I, restriction enzymes sites. **(B)** Summary of 5' deletion constructs of the *N. benthamiana* polymerase I promoter. Arrows denote positions at which transcripts are initiated (defined as +1). **(C)** Schematic representation of the agroinfiltrated zones in *N. benthamiana* leaf. **(D)** GFP expression pattern (under UV light) of agroinfiltrated *N. benthamiana* leaves at 4, 6, and 8 dpi, respectively. **(E)** SDS-PAGE analysis and immunodetection of proteins isolated from the agroinfiltrated zones of *N. benthamiana* leaves. The gel was stained with Coomassie brilliant blue (upper panel) and the corresponding antibodies were used to detect GFP (lower panel) in agroinfiltrated *N. benthamiana* leaves by Western blots. M, molecular weight marker (kDa); H, total soluble protein extracts from uninoculated leaves; 498, 251, 125, and 77, total soluble protein extracts from the corresponding agroinfiltrated zones of *N. benthamiana* leaves in **(C)**. **(F)** Time course for the accumulation of recombinant GFP protein in *N. benthamiana* leaves by ELISA.

To analyze the organization of the *Nb*rRNA gene promoter, successive 5'-promoter deletions were prepared and designated as −498, −251, −125, and −77 (Figures 1A,B). These promoter deletions were used to substitute the 35S promoter of the Pol II-G vector (Figure 1A), which is a TBSV-based viral vector carrying the reporter gene of GFP. After delivery into *N. benthamiana* plants *via* the *Agrobacterium*-mediated method, the constructs were transcribed to generate the viral genomic mRNA by the tested promoters, and then the recombinant GFP proteins were translated in the host cell from the viral sg mRNA1. The expressed GFP was readily detected either by the observation of its displayed fluorescence or immunoassays. The differences in GFP expression may reflect differences in transcript abundance driven by the tested promoter.

Agroinfiltration zones for each inoculum are presented in Figure 1C. The infiltrated leaves were visualized under UV light for GFP expression at 4–8 dpi. The green fluorescence was observed at infiltrated zones with all 5'-deletion promoter constructs (Figure 1D), indicating the *Nb*rRNA gene promoter can drive viral RNA-based vector to express GFP in *N. benthamiana* and this viral RNA-based reporter system could be used to measure rRNA gene promoter activity in plants. The fluorescence signals increased gradually over time within the infiltrated area. Replication of the TBSV reporter replicon may explain this phenomenon. Besides that, there was no noticeable difference in the intensity of GFP fluorescence from all four constructs. GFP expression was subsequently confirmed by the existence of an approximately 27.0-kDa protein band in both SDS-PAGE and Western blots assay (Figure 1E) and the accumulation of recombinant GFP was quantified using GFP ELISA (Figure 1F). These results indicate that sequences between −489 and −78 may play a minor role in *Nb*rRNA gene promoter activity because all deletion promoter constructs reproducibly yielded the almost same amount of recombinant GFP (Figure 1F). Similarly, it has been noted that the 5' sequences (between −2,590 and −92) of the *A. thaliana* rRNA gene promoter make only a small contribution to the promoter activity (Doelling et al., 1993). This differs from what is obtained from animals, in which around a 15-fold decrease in promoter activity is observed upon deletion of the UCE (usually spans the region from −186 to −107; Clos et al., 1986; Haltiner et al., 1986).

### Effect of the Sequences Located Downstream of TIS on *Nb*rRNA Gene Promoter Activity

**Figure 2:**
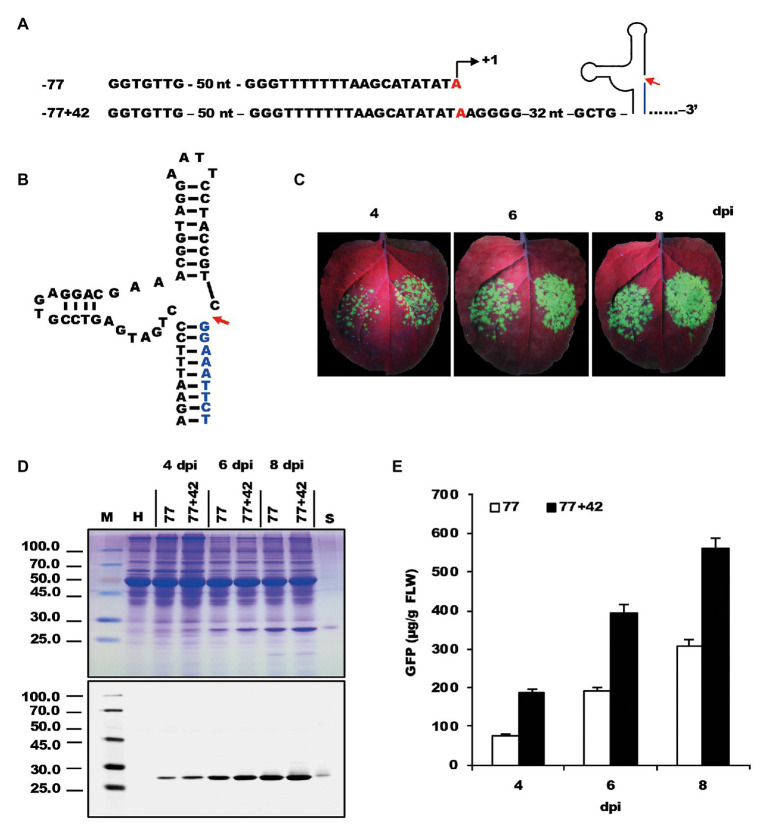
Testing the effect of the sequence downstream of transcription start site on the expression of recombinant proteins. **(A)** The nucleic acid sequences of mini *Nb*rRNA gene promoter (−77) and its analog with downstream extension sequences and a ribozyme sequence. **(B)** The nucleic acid sequences and structure of the hammerhead ribozyme. **(C)** GFP expression pattern (under UV light) of agroinfiltrated *N. benthamiana* leaves at 4, 6, and 8 dpi, respectively. The left side of the leaf was agroinfiltrated with a −77 vector and the right side was −77+42. **(D)** SDS-PAGE analysis and immunodetection of proteins isolated from the agroinfiltrated zones of *N. benthamiana* leaves. The gel was stained with Coomassie brilliant blue (upper panel) and the corresponding antibodies were used to detect GFP (lower panel) in agroinfiltrated *N. benthamiana* leaves by Western blots. M, molecular weight marker (kDa); H, total soluble protein extracts from uninoculated leaves; 77 and 77+42, total soluble protein extracts from the corresponding agroinfiltrated zones of *N. benthamiana* leaves in **(C)**. **(E)** Time course for the accumulation of recombinant GFP protein in *N. benthamiana* leaves by ELISA.

To determine whether the sequence downstream of TIS is also involved in transcription, the −77 *Nb*rRNA gene promoter was extended downstream to include another 42-nt-long sequence (Figure 2A). A hammerhead ribozyme sequence (Figure 2B) was placed between the “extended +42” promoter and viral sequence to maintain the 5'-end integrity of transcribed viral genomic RNA. The generated −77+42 construct was then agroinoculated on the right side of *N. benthamiana* leaf along with the −77 construct on the left side as a control.

Observation of infiltrated tissue under UV light demonstrated that the fluorescence of the inoculated zones of the −77 construct was less than that of the −77+42 construct, showing a significant increase in GFP expression along with the −77+42 construct (Figure 2C). Data analysis of SDS-PAGE and Western blots supported the fluorescence observations (Figure 2D). The relative level of GFP expression revealed a 2–3-fold increase with TIS downstream extension (Figure 2E). The results suggest that the sequences located downstream of TIS affects promoter strength and is an essential element of the *Nb*rRNA gene promoter. This conclusion is in agreement with a previous study (Doelling et al., 1993), which provided evidence that the 3' boundary of the *A. thaliana* rRNA gene promoter is determined to be within the interval −6 to +6.

### Effect of the Heterologous Enhancers on *Nb*rRNA Gene Promoter Activity

Doelling and Pikaard (1996) speculated that the *Brassica oleracea* rRNA gene promoter was only about one-tenth of the strength of the CaMV-35S promoter. In our previous study, the amount of GFP expressed from the −498 *Nb*rRNA gene promoter was indeed lower when compared to a 35S promoter-controlled construct in the same experiments (data not shown). Schultz et al. (1993) reported that an *Eco*RI-*Hpa*I fragment of the *Saccharomyces cerevisiae* ribosomal gene spacer can enhance transcription from an adjacent RNA polymerase I promoter *in vitro*. To explore whether the heterologous enhancer could increase the activity of the *Nb*rRNA gene promoter, the *Eco*RI-*Hpa*I fragment and the enhancer of 35S promoter (−343 to −90) were used to generate two enhancer-containing constructs, E1−77+42 and E2−77+42.

**Figure 3:**
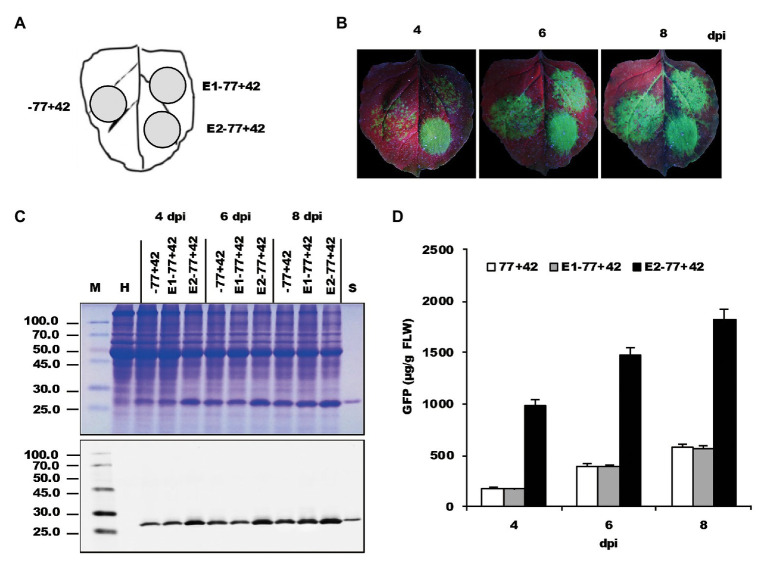
The effects of two enhancers on the expression of recombinant proteins. **(A)** Schematic representation of the agroinfiltrated zones in *N. benthamiana* leaf. **(B)** GFP expression pattern (under UV light) of agroinfiltrated *N. benthamiana* leaves at 4, 6, and 8 dpi, respectively. **(C)** SDS-PAGE analysis and immunodetection of proteins isolated from the agroinfiltrated zones of *N. benthamiana* leaves. The gel was stained with Coomassie brilliant blue (upper panel) and the corresponding antibodies were used to detect GFP (lower panel) in agroinfiltrated *N. benthamiana* leaves by Western blots. M, molecular weight marker (kDa); H, total soluble protein extracts from uninoculated leaves; −77+42, E1−77+42 and E2−77+42, total soluble protein extracts from the corresponding agroinfiltrated zones of *N. benthamiana* leaves in **(A)**. **(D)** Time course for the accumulation of recombinant GFP protein in *N. benthamiana* leaves by ELISA.

After inoculation on the *N. benthamiana* leaves (Figure 3A), GFP fluorescence was easily observed in the area infiltrated with bacterial suspensions containing various constructs (Figure 3B). Interestingly, a notable difference in fluorescence strength was observed between the zones inoculated with E2−77+42 and −77+42. However, it did not reveal a significant difference between the E1−77+42 and −77+42. The result was confirmed by immunoblot analysis (Figure 3C) and ELISA assay (Figure 3D), which showed an approximately 4–5-fold increase by the 35S enhancer at 4 dpi. Those data indicated that *Nb*rRNA gene promoter activity might be improved by an appropriate heterologous enhancer sequence.

### The Transcription Specificity of the *Nb*rRNA Gene Promoter

**Figure 4:**
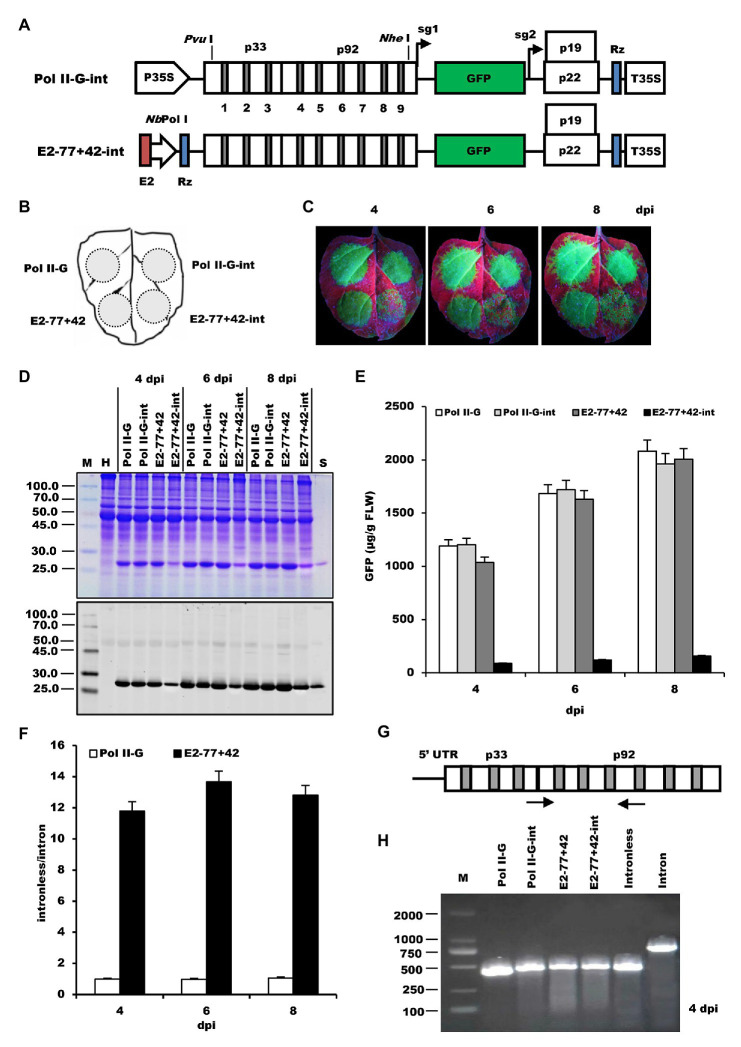
Testing the transcription specificity of the *Nicotiana benthamiana* rRNA (*Nb*rRNA) gene promoter by an intron-excision mediated assay. **(A)** Schematic representation of the intron-containing Pol I-promoter- or Pol II-promoter-based TBSV vectors. P35S, 35S promoter; p33 and p92, the gene coding replication proteins; sg1 and sg2, the TIS for subgenomic mRNAs 1 and 2 indicated by right-angled arrows; green fluorescent protein (GFP), enhanced green fluorescent protein gene; p19, silencing suppressor gene; p22, cell-to-cell movement protein (MP) gene; Rz, ribozyme; T35S, cauliflower mosaic virus (CaMV) polyA signal sequence/terminator; *Pvu* I and *Nde* I, restriction enzymes sites; E2, ELS from enhanced 35S promoter; *Nb*Pol I, modified *Nb*rRNA gene promoter (−77+42). The light gray box in the p33 and p92 regions shows the positions of the different introns. **(B)** Schematic representation of the infiltrated zones in *N. benthamiana* leaf. **(C)** GFP expression pattern (under UV light) of agroinfiltrated leaves at 4, 6, and 8 days post-inoculation (dpi), respectively. **(D)** SDS-PAGE analysis and immunodetection of proteins isolated from the agroinfiltrated leaves. The gel was stained with Coomassie brilliant blue (upper panel) and the corresponding antibodies were used to detect GFP (lower panel) by Western blots. M, molecular weight marker (kDa); H, Protein extracts from uninoculated leaves; Pol II-G, Pol II-G-int, E2−77+42, and E2−77+42-int, protein extracts from the corresponding infiltrated zones of *N. benthamiana* leaves in **(C)**. **(E)** Time course for the accumulation of GFP in infiltrated leaves by ELISA. **(F)** The ratios of GFP accumulation between intronless/intron-containing constructs. **(G)** The map of introns in the p33 and p92 region and the position of their reverse transcription PCR (RT-PCR) primers. **(H)** The RT-PCR analysis of introns in p33 and p92 at 4 dpi. Lane M: DNA molecular standards (bp); Pol II-G, Pol II-G-int, E2−77+42, and E2−77+42-int, total RNAs from the corresponding infiltrated zones of leaves in **(C)**; Intronless, synthesized p33 and p92 sequences without introns; Intron, synthesized p33 and p92 sequences with introns.

To test the transcription specificity of the *Nb*rRNA gene promoter, an intron-excision mediated assay was established. Two constructs, Pol II-G-int, and E2−77+42-int, which are under the control of either Pol II or Pol I promoter and contain nine *A. thaliana* introns in the viral RdRp coding region, were created, respectively, (Figure 4A). Theoretically, during transcription driven by Pol II, these introns will be excised to produce a functional virus mRNA, which then synthesize the proteins of the viral replicase. However, if the introns are not removed correctly during splicing, no viral RNA transcription and replication will occur because of translational frameshift in the replicase gene. In contrast, the products transcribed by Pol I do not include splicing, so intron insertion into the viral genomic mRNA is expected to abolish the protein synthesis (Komarova et al., 2012).

*Nicotiana benthamiana* leaf was agroinoculated with *Agrobacterium* cultures carrying these two constructs along with its corresponding intronless constructs as control (Figure 4B). At 4–8 dpi, the strong green fluorescence was seen throughout the zones inoculated with Pol II-G, Pol II-G-int, and E2−77+42, but there were only dispersed green spots showing on the zone inoculated with E2−77+42-int (Figure 4C). Western blot analysis and ELISA assay also revealed the dramatic decrease of GFP expression of E2−77+42-int (Figures 4D,E). Remarkably, E2−77+42-int, an intron-containing construct driven by Pol I promoter, still expressed the recombinant GFP proteins in the inoculated leaves at a relatively low level and the ratio of GFP expression between the intronless and intron-containing construct was about 12–15: 1 (Figure 4F). Comparably, the ratio of the two Pol II promoter constructs was close to 1 (Figure 4F). Reverse transcription PCR (RT-PCR) results demonstrated that the introns in the E2−77+42-int construct were removed by nuclear processing machinery by 4 dpi (Figures 4G,H). Doelling and Pikaard (1996) showed that the *Arabidopsis* rRNA gene promoter could be used inefficiently by a different polymerase in *Arabidopsis* cells (Doelling and Pikaard, 1996). Therefore, we speculated that the RNA polymerase II might participate in the Pol I promoter-mediated transcription.

**Figure 5:**
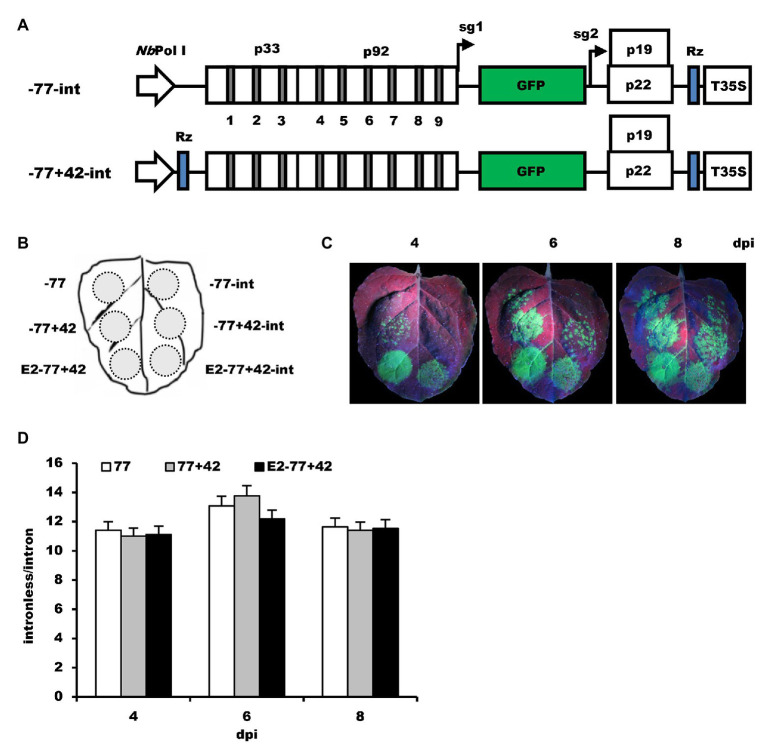
The effects of modification of *Nb*rRNA gene promoter on its transcription specificity. **(A)** Schematic representation of the intron-containing Pol I-promoter-based TBSV vectors. -77-int, mini *Nb*rRNA gene promoter (−77)-based TBSV vector with introns; −77+42-int, TBSV vector containing introns under control of mini *Nb*rRNA gene promoter (−77) with downstream extension sequences and a ribozyme sequence. **(B)** Schematic representation of the agroinfiltrated zones in *N. benthamiana* leaf. **(C)** GFP expression pattern (under UV light) of agroinfiltrated *N. benthamiana* leaves at 4, 6, and 8 dpi, respectively. **(D)** The ratios of GFP accumulation between intronless/intron-containing constructs.

To examine if the modification of the *Nb*rRNA gene promoter results in the non-specificity of transcription, two other intron-containing constructs, −77-int and −77+42-int (Figure 5A), were generated based on −77 and −77+42. After inoculation on the *N. benthamiana* leaves (Figure 5B), GFP fluorescence was observed (Figure 5C), and the leaf samples collected from the infiltrated area were analyzed by ELISA assay (Figure 5D). The data showed that even the non-modified *Nb*rRNA gene promoter (−77) construct with introns still can express GFP proteins and the ratio of GFP expression between the intronless and intron-containing construct is similar in all tests, indicating that the non-specificity of *Nb*rRNA gene promoter transcription is not due to the modification and it is the intrinsic characteristic for *Nb*rRNA gene promoter.

### Improving Transcription Specificity of the *Nb*rRNA Gene Promoter by Mutation

**Figure 6:**
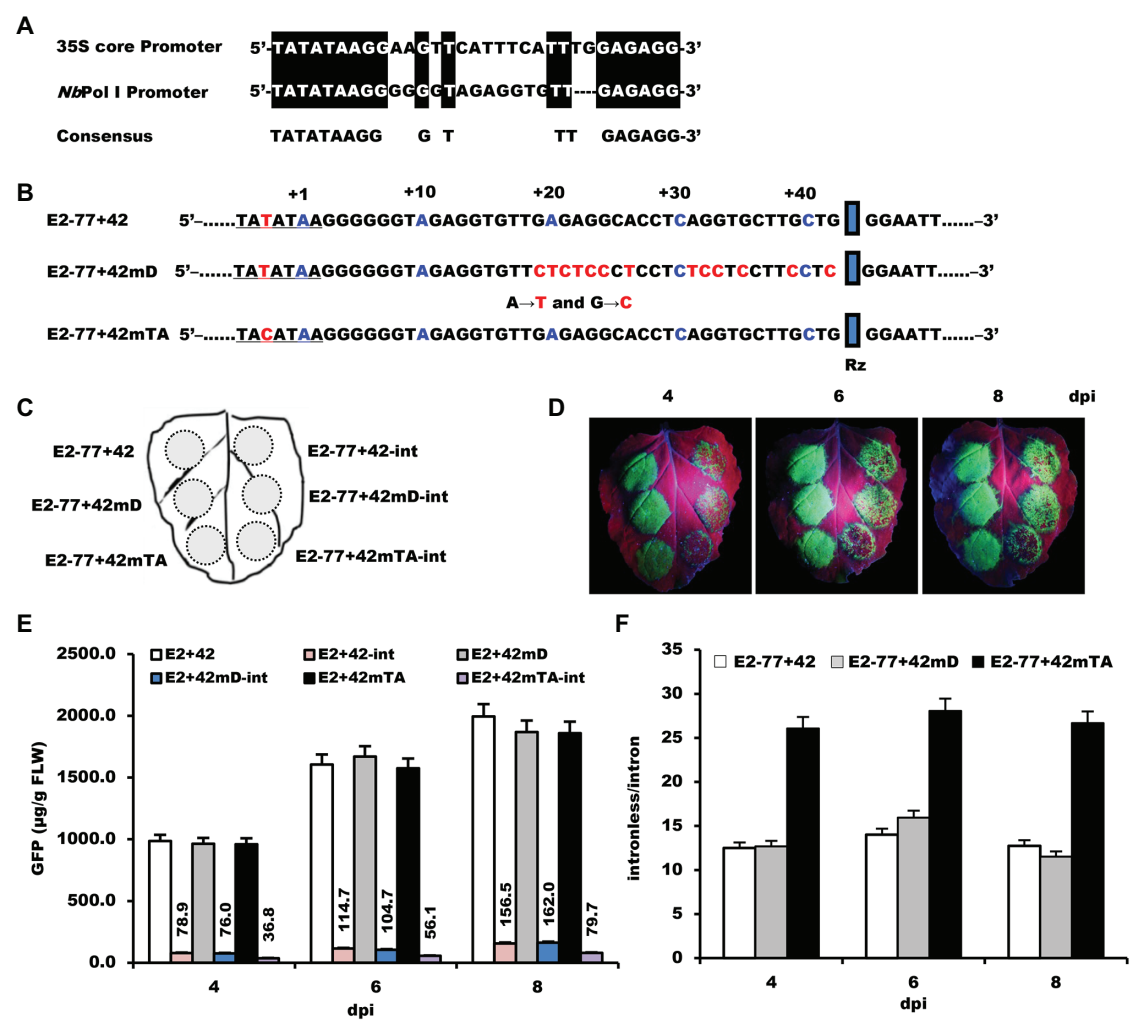
The effects of mutation of *Nb*rRNA core promoter on its transcription specificity. **(A)** Alignment of 35S and Pol I core promoter. **(B)** The nucleic acid sequences and the mutation position of the E2−77+42 promoter. **(C)** Schematic representation of the agroinfiltrated zones in *N. benthamiana* leaf. **(D)** GFP expression pattern (under UV light) of agroinfiltrated *N. benthamiana* leaves at 4, 6, and 8 dpi, respectively. **(E)** Time course for the accumulation of GFP in infiltrated leaves by ELISA. **(F)** The ratios of GFP accumulation between intronless/intron-containing constructs.

Usually, plant RNA polymerase II core promoter includes either a TATA-box and/or an initiator (Inr) element. The transcriptional Inr overlaps the TIS and locates about 25–30 bp downstream of the TATA-box (Shahmuradov et al., 2003). Transcriptional activation requires the TATA-box element, and the initiator element is responsible for the precise location of the start site (Sainsbury et al., 2015). Comparison of the core sequence of Pol II promoter (35S) and *Nb*rRNA gene promoter regions show that both promoters share a consensus TATA-like sequence and the relatively conservative downstream flanking sequences (Figure 6A), indicating that RNA polymerase II may be able to recognize the *Nb*rRNA gene promoter. Theoretically, mutation of these two *cis*-elements may influence the transcription activities of the Pol II promoter, decreasing the opportunity of RNA polymerase II-dependent transcription.

For the initiator element, purine-to-pyrimidine conversion at the +1 site affected the selection of the TIS of RNA polymerase II-dependent transcription (Zhu et al., 1995). Therefore, the *Nb*rRNA gene promoter sequence, which is located 15–42 bp downstream of the TATA-like sequence, was substituted by A–T and G–C to generate construct E2−77+42 mD (Figure 6B). For the TATA-box element, even a single nucleotide mutation can remarkably reduce the transcriptional efficiency of RNA polymerase II-dependent transcription (Sainsbury et al., 2015). While searching the *N. benthamiana* genome database, we found one of the *Nb*rRNA gene promoter sequences contains a T–C transition at −3 in the TATA-like sequence. To see the effect of this nucleotide change, we substituted T for C at −3 in the TATA-like region to generate construct E2−77+42mTA (Figure 6B).

The constructs and their corresponding intron versions were inoculated on the *N. benthamiana* leaf as shown in Figure 6C. The intron version of E2−77+42mTA (E2−77+42mTA-int) showed fewer green spots on its inoculation zone (Figure 6D) than E2−77+42-int and E2−77+42 mD-int, which showed similar fluorescence signals. ELISA assay revealed that the ratio of GFP accumulation differed by 2-fold between E2−77+42mTA-int and E2−77+42-int (Figures 6E,F). Moreover, this single nucleotide mutation did not affect the transcription activities of the *Nb*rRNA gene promoter as the E2−77+42 and E2−77+42mTA had the same accumulation level of GFP (Figure 6E). These results suggested that the mutation of the TATA-like sequence can improve the transcription specificity of the *Nb*rRNA gene promoter. It also provided indirect evidence that the *Nb*rRNA gene promoter was inefficiently utilized by RNA polymerase II in *N. benthamiana* cells.

## Discussion

Reporter gene-based expression systems have been intensively used in plants for studying the activity of gene promoters. However, the plant rRNA transcripts are not capped, unlike mRNAs, thus they cannot be engaged by ribosomes and translated efficiently. To conquer this “bottleneck,” it is necessary to circumvent the need for a cap of Pol I transcripts. Palmer et al. (1993) showed that Pol I transcription can be used to express protein-coding reporter genes in mammalian cells by using viral IRES sequences. In this study, we used the plant virus RNA-derived sequences to avoid the need for a cap.

The plant virus genomic RNAs are some of the most efficiently translatable mRNAs (Fan et al., 2012). Like the products transcribed by RNA polymerase I, many plant viral RNAs lack a 5' cap structure (Kneller et al., 2006); but unlike RNA polymerase I transcripts, these uncapped RNAs are translated efficiently *via* a non-canonical mechanism (Firth and Brierley, 2012). Among the known plant RNA viruses with uncapped genomic RNAs, TBSV contains only one small genomic RNA segment that makes it much easier for genetic manipulation (White and Nagy, 2004). Moreover, a TBSV-CP replacement vector has been developed to transiently express reporter genes in various plant species through agroinfiltration (Shamekova et al., 2014; Zhang et al., 2020). And those TBSV-based constructs replicate efficiently in inoculated plant leaves (Zhang et al., 2020). Thus, it is a good candidate for establishing a transient assay approach for analyzing the activity of the rRNA gene promoter.

Here, we developed a reporter system to analyze the characteristic of the *N. benthamiana* rRNA gene promoter. The system uses viral replication to amplify the rRNA transcript signal and viral mRNA-expressed GFP as an indicator of rRNA gene promoter activity in virus-infected plants. The GFP fluorescence is easily observed on the infiltration of *N. benthamiana* leaves (Figure 1D) and the amount of GFP can be qualitatively or quantitatively detected by traditional Western blots (Figure 1E) or ELISA (Figure 1F). We examined the functional architecture of the *Nb*rRNA gene promoter by analysis of the GFP expression from extracts of inoculated *N. benthamiana* leaves. Some of our results are consistent with what was obtained from previous studies on the *Arabidopsis* rRNA gene promoter (Doelling et al., 1993), demonstrating that this system has a good potential for the rRNA gene promoter assay. It is easier to operate and more convenient, while compared with the previously rRNA gene promoter assays.

It may be argued that upon transient expression, the transcription of foreign DNA is not performed in Nucleolar Organizer Regions (NORs) and/or in the nucleolus, instead, rDNAs are located in NORs and are transcribed in the nucleolus. The nucleolus does not have a known membrane and/or other physical barriers (Scheer and Weisenberger, 1994) and all three nuclear RNA polymerases are not separated from each other within the nucleus (Doelling and Pikaard, 1996). An ectopic-integrated rRNA gene can also be transcribed by RNA polymerase I (Johnson and Warner, 1989; Karpen et al., 1989). Moreover, a mini-nucleolus is assembled around the ectopic rRNA gene in *Drosophila* (Karpen et al., 1989), indicating that the nucleolus comes about as a result of the rRNA gene expression instead of a prerequisite.

In our study, the 5' boundary of *N. benthamiana* rRNA gene promoter was defined at least as far as −77 without losing promoter activity. However, we did not further define the 5' boundary of *N. benthamiana* rRNA gene promoter. It has been shown that diploid wheat rRNA gene promoter sequences localized between −113 and +15 are sufficient to guide RNA Pol I-mediated transcription (Akhunov et al., 2001) and the 5' boundary of *A. thaliana* rRNA gene promoter located between −55 and −33 (Doelling and Pikaard, 1995). Notably, the plant rRNA gene promoters are a little more conserved between −55 and +1. Therefore, the minimal 5' boundary of the plant rRNA gene promoter could be less than −55. Moreover, the 5' sequences of the *N. benthamiana* rRNA gene promoter between −498 and −77 have no significant effects on promoter activity, indicating that there are no distinct functional domains upstream of −77. Similar results were obtained from *A. thaliana* (Doelling and Pikaard, 1995) and diploid wheat (Jackson and Flavell, 1992), where deletion of the 5' flanking sequences to −55 or −133 did not significantly influence transcription activity in transfected protoplasts, suggesting that the organization of plant rRNA gene promoter differ from those of animal or fungi. Interestingly, the sequence located downstream of the TIS of the *Nb*rRNA gene promoter is also required for efficient transcription. The DNase I studies revealed that the *Brassica* rRNA promoter region between −30 and +20 was protected from DNase I digestion by RNA polymerase I (Saez-Vasquez and Pikaard, 2000). The wheat rRNA promoter region from −34 to +10 was proved to be bound by isolated nuclear proteins *in vitro* (Akhunov et al., 2001). Also, point mutations in the region from −6 to +5 of the *Arabidopsis* rRNA promoter influenced promoter strengths (Doelling and Pikaard, 1995). Our construct E2−77+42 mD, in which mutations were made from +18 to +42, gave no detectable effect on promoter activity (Figure 6), indicating that the sequence downstream of +18 is not likely necessary for promoter activity. The above-mentioned evidence suggests that the sequences near downstream of the TIS of the rRNA gene promotor might participate in the interaction with RNA polymerase I or auxiliary cofactors. That also might explain an increase of promoter activity by the extension of the TIS downstream sequences in our study.

In *Arabidopsis*, it has been reported that RNA polymerase II can recognize the rRNA promoter and initiate transcription from an altered transcription start site (Doelling and Pikaard, 1996). However, Fan et al. (1995) showed that crude extracts from tobacco cells do not activate the transcription of the broad bean rRNA genes *in vitro*, indicting species-specificity of rDNA transcription in plants. Therefore, an intron-excision mediated assay was established to test the transcription specificity of the *Nb*rRNA gene promoter. Due to the difference of post-transcriptional processing between Pol II and Pol I, it is possible to differentiate one type of polymerase activity from another by this assay. One may argue that alpha-amanitin treatment also can be used to distinguish the two polymerases mediated transcription because they have different sensitivity to alpha-amanitin (Jendrisak and Guilfoyle, 1978). Unfortunately, the whole plant or protoplast is not sensitive to alpha-amanitin treatment (Doelling and Pikaard, 1995). Like what was obtained from *Arabidopsis*, our results also suggest that in *N. benthamiana*, the RNA polymerase II may recognize the rRNA gene promoter and inefficiently initiate transcription. This conclusion is drawn from three pieces of evidence. First, the core sequence between the Pol II promoter (35S) and the *Nb*rRNA gene promoter is highly conserved (Figure 6A). Second, intron-containing constructs driven by the rRNA gene promoter still expressed the reporter gene in the inoculated leaves (Figures 4, 5). Third, a single nucleotide mutation in the TATA-like box did decrease the expression level of the reporter gene by rRNA gene promoter-driven intron-containing constructs (Figure 6). Doelling and Pikaard (1996) speculated that switching in RNA polymerase could give plant cells more regulatory plasticity to produce rRNAs. Moreover, this characteristic differs from the animal rRNA gene promoter, where the promoter activity is highly species-specific (Utt et al., 2019).

Enhancer elements were well characterized for RNA polymerase II genes (Andersson and Sandelin, 2020). However, enhancer elements, which can stimulate RNA polymerase I transcription, have been described only for the rRNA genes of *Xenopus* spp. (Labhart and Reeder, 1985), *S. cerevisiae* (Johnson and Warner, 1989), mouse (Pikaard et al., 1990), and so on. Furthermore, most of the cloned RNA polymerase I enhancers show trans-species or trans-kingdom activities. For instance, a cloned mouse enhancer can boost frog rRNA gene transcription in injected frog oocytes (Pikaard et al., 1990). Likewise, the cloned *Arabidopsis* enhancer can increase the transcription level of the frog rRNA gene in injected *Xenopus* oocytes (Doelling et al., 1993). In our study, the accumulation of recombinant GFP was increased by adding the enhancer of the 35S promoter, instead of yeast rDNA enhancer. It may be argued that the GFP increase was due to the stimulation of Pol II transcriptions by the 35S enhancer because of a switch in RNA polymerase specificity. Based on the theory of our intron-excision mediated method, the intron-containing virus constructs are responsible for the GFP expression mediated by Pol II and the intronless constructs might be responsible for the accumulation of both Pol I- and Pol II-mediated GFP expression. The ratio of GFP accumulation differed by 2-fold between the intron-containing constructs E2−77+42-int and E2−77+42mTA-int (Figure 6F). However, the accumulation level of GFP is similar between the intronless constructs E2−77+42 and E2−77+42mTA (Figure 6E). The Pol II transcriptions cannot be responsible for all the increase of GFP expression, which was stimulated by enhancer sequences. Therefore, the 35S enhancer might stimulate the transcription of Pol I promoters. Although only a 4–5-fold increase was achieved by the 35S enhancer (Figure 3D), conversely, a 10–200-fold increase obtained by the enhancers for RNA polymerase II genes (Andersson and Sandelin, 2020), it is the first evidence that the RNA Pol II gene enhancer can stimulate the transcription of plant rRNA gene promoter. Certainly, it still needs more experiments to figure out whether the 35S enhancer can also stimulate the transcription of the rRNA gene promoter isolated from other species.

One might expect that the high efficiency of virus replication may obscure the differences in Pol I promoter activity. Very low levels of an initial transcript may be amplified to a level that reduces the differences in initial transcript levels from the promoter. So, it is possible that the 15-fold decrease in GFP caused by intron insertion, or the 4–5-fold increase due to the 35S enhancer, might be more than suggested by GFP levels. Even so, the TBSV replicon offers more sensitivity, and indeed shows some quantitative response that makes it a useful system.

## Conclusion

Plant rRNA gene promoters are less well characterized, largely due to the lack of a convenient and robust assay system to measure the promoter activity. In this study, we established a viral RNA-based reporter assay to characterize the rRNA gene promoter of *N. benthamiana*. The developed assay system provides a novel solution to rRNA gene promoter analysis by employing a cap-independent translation strategy of viral RNAs. Besides, rRNA gene promoters of other plant species can be investigated by this methodology, because of the availability of the diverse viral RNA-based vectors. Furthermore, the system can also be used for the protoplast system by directly transferring virus plasmid vectors. Taken together, this RNA virus-based assay system could open up new possibilities for analyzing the characteristic of plant rRNA gene promoters.

## Data Availability Statement

The raw data supporting the conclusions of this article will be made available by the authors, without undue reservation.

## Author Contributions

SW conceived, designed, and supervised the study, discussed the results, and wrote the manuscript. LX and ZL performed the experiments, analyzed the data, and wrote the manuscript. All authors contributed to the article and approved the submitted version.

### Conflict of Interest

The authors declare that the research was conducted in the absence of any commercial or financial relationships that could be construed as a potential conflict of interest.
